# Supplementary material for: Bacterial fumarase and L-malic acid are evolutionary ancient components of the DNA damage response
Source: eLife. 2017 Nov 15;6:e30927. doi: 10.7554/eLife.30927 (PMC5711358; doi:10.7554/eLife.30927)
Supplement: Supplementary file 1. — B. subtilis strains used in this study and their source. [file elife-30927-supp1.docx]

Table S1

| Strain | Genotype | Comments |
| --- | --- | --- |
| PY79 | *Wild type* | (Youngman et al., 1984)[[37](#_ENREF_34)] |
| *Δfum* | *citG:: kan* | The ORF of citG was replaced by kan gene using a long-flanking-homology PCR with primers **A-5’** TCC TGG ATA TCT TCC AGC TGG AT **B-5’** ATC ACC TCA AAT GGT TCG CTG GGT TT TTA TGT ATC CCT CCA TAA CGG TTG **C-5’** AAG TTC GCT AGA TAG GGG TCC CGA GC GCG TAA TAG GAA GAA CGG CTG CTT **D-5’** AAC AGA CGG TGT GAC TAT ATG GTC |
| *Δfum+fum-bc* | *citG::kan,*  *amyE:: citG-spc* | Δfum was transformed with Pfum-bc (amyE:: citG spc) |
| Fum-GFP | citG-gfp-spc | (Lemon and Grossman, 1998)[[38](#_ENREF_35)] |
| H186N | *citG: H186N* | PY79 was transformed with PH168N |
| H127R | *citG: H127R* | PY79 was transformed with PH127R |
| RecN-GFP | *RecN-GFP* | Kindly provided by ben-yehudas lab |
| RecN-GFP, *Δfum* | *RecN-GFP, citG:: kan* | RecN-GFP was transformed with Δfum strain |
| *ΔRecN* | *recN::Cm* | Kindly provided by Alonso lab |
| *Δfum, ΔRecN* | *recN::Cm, citG:: kan* | ΔRecN was transformed with Δfum |
