# Supplementary material for: Bacterial fumarase and L-malic acid are evolutionary ancient components of the DNA damage response
Source: eLife. 2017 Nov 15;6:e30927. doi: 10.7554/eLife.30927 (PMC5711358; doi:10.7554/eLife.30927)
Supplement: Supplementary file 2. — S. cerevisiae strains used in this study and their source. [file elife-30927-supp2.docx]

Table S2

| Strain | Genotype | Comments |
| --- | --- | --- |
| Sc WT (BY4741) | Mat a; his3D1; leu2D0;  met15D0; ura3D0 |  |
| *Δfum1* |  | Sass E et al., 2003 [[18](#_ENREF_36)] |
| *FUM1m* |  | Yogev O et al., 2010[[3](#_ENREF_3)] |
